# Risk Factors for Deterioration of the Clinical Condition of Cull Dairy Cows During Transport to Slaughter

**DOI:** 10.3389/fvets.2018.00297

**Published:** 2018-11-22

**Authors:** Kirstin Dahl-Pedersen, Mette S. Herskin, Hans Houe, Peter T. Thomsen

**Affiliations:** ^1^Department of Animal Science, Aarhus University, Tjele, Denmark; ^2^Department of Veterinary and Animal Sciences, University of Copenhagen, Frederiksberg C, Denmark

**Keywords:** dairy cows, animal welfare, fitness for transport, lameness, animal transportation

## Abstract

Cull dairy cows are typically transported to slaughter by road. Across different types of cattle, road transport is recognized as stressful. Cull dairy cows may have different injuries or weaknesses and may thus be more vulnerable to transport stress than other types of cattle. The aim of this study was to investigate whether the clinical condition of cull dairy cows deteriorates during transport (< 8 h), and to evaluate risk factors for potential deterioration of the clinical condition. A total of 411 dairy cows were clinically examined on farm before loading and again after unloading at the slaughter plant. The clinical examination included locomotion, presence of wounds, milk leakage, and general condition. One-fifth of the cows either became lame or more lame during transport, and there was a significant increase in the proportion of lame cows after transport (41% after vs. 31% before, *P* < 0.0001). A significant increase in the proportion of cows with milk leakage (17% vs. 1%, *P* < 0.0001) and wounds (34% after vs. 22% before, *P* < 0.0001) after transport were also found. Low body condition score (BCS) (< 2.75) (*P* = 0.001), early or late lactation [< 100 days in milk (DIM) or >300 DIM] (*P* = 0.01), digital dermatitis in the hind feet (*P* = 0.01), and pelvic asymmetry (*P* = 0.001) were identified as risk factors for the deterioration in lameness during transport. Early lactation (< 100 DIM) (*P* = 0.04) and transport distance (>100 km) (*P* = 0.006) were identified as risk factors for milk leakage. For wounds, no significant risk factors were found. The results demonstrate that cull dairy cows are vulnerable to the strains of transport, even journeys shorter than 8 h, to the extent that the occurrence of clinical findings were increased after transport in cows legally considered fit for transport. These results call for further research into the animal welfare implications and optimization of cattle transport.

## Introduction

Cull dairy cows are typically transported to slaughter by road, either directly or through markets (1). Across countries, recently reported proportions of the milking herd that is culled annually range from 23 to 36% (2–4), which means that worldwide millions of cull dairy cows are transported to slaughter each year.

Dairy cows are culled for a number of reasons relating mainly to productivity and health (5–7) and in many cases more than one culling reason are reported by farmers (8). Recently, we presented data from clinical examinations of the cows involved in the present study—while they were still on-farm before transport (9)—showing that almost 75% of the cows deviated clinically from the normal condition. Overall, the cull dairy cows constituted a diverse group of animals in terms of age, parity, milk yield, type, and number of clinical signs.

Across different types of cattle, road transport is recognized as stressful [as reviewed by Knowles (10)]; however only few studies have included cows (11–15). Based on the knowledge that dairy cows are often culled due to clinical conditions or weaknesses, such as mastitis or lameness (5–7) as well as the relatively high proportion of cows characterized by clinical deviations from normal conditions (9), it seems justified to suggest that dairy cows are more vulnerable to transport stress than other types of cattle [as suggested by Nielsen et al. (16)]. In accordance with this, Gonzalez et al. (12) found that cull cows were at a higher risk of becoming non-ambulatory during long haul transport than fattened cattle. Brown et al. (11) investigated the incidence of dark cutting beef; a well-described meat quality indicator associated with pre-slaughter stress, and found higher incidences for cull cows than for steers, heifers, or calves. In addition, Malena et al. (13) found that dairy cows had a higher risk of dying during transport than fattened cattle or calves sent to slaughter. Although these results were not based on direct comparison of cull dairy cows with other types of cattle transported under identical conditions, they may indicate that cull dairy cows tolerate transport less well than fattened cattle.

However, only few studies have described the clinical condition of cull cows after transport. Ahola et al. (17) did a survey of cattle sold through markets immediately before slaughter and found 13% of dairy cows to be emaciated or near emaciated and 45% to be lame. Nicholson et al. (14) inspected dairy cows as they arrived at slaughter plants and found 5% to be emaciated and 49% to be lame. Rezac et al. (18) and Strappini et al. (19) examined cull cow carcasses and found 54 and 92%, respectively, to be bruised. However, in order to understand the potential changes in the clinical condition after transport, it is necessary to gather information of the clinical condition both before and after transport, which was not included in the above-mentioned studies.

The European Council Regulation on animal transport [EU Regulation, EC 1/2005 (20)] is, in general, precise, and objective for instance regarding loading densities or journey durations. One exception, however, is the rules on fitness for transport, which are vaguely defined and lack clear cut-off points [as reviewed by Grandin (21) for cull sows]. The EU regulation [EC 2005/1 (20)] clearly states that animals must be fit for the journey in order to be transported and may not be transported in a way that will injure them or cause undue suffering. However, the regulation also states that animals that are “slightly ill or injured” and will not experience additional suffering due to transport may be considered fit for transport. How to assess “slightly ill or injured” and “additional suffering” is not defined, but a few severe conditions that would define an animal as unfit for transport are listed in the regulation, e.g., inability to walk unassisted, severe open wounds, or prolapses. Cull animals are not mentioned specifically in the regulation. Hence, knowledge about potential deterioration of the clinical condition in cull dairy cows during transport will add significantly to this area.

The objectives of this study were to (1) investigate whether the clinical condition of cull dairy cows changes during transport to slaughter; and (2) evaluate risk factors for potential deterioration of the clinical condition. In order to do so, we performed clinical examinations of 411 cull dairy cows before and after transport to a slaughter plant with special focus on locomotion, presence of wounds, milk leakage, and general condition. We hypothesized that a potential deterioration of the clinical condition would be associated with (1) distance and duration of transport; (2) clinical weaknesses already present before transport e.g., lameness or mastitis; (3) production related factors e.g., parity or days in milk (DIM).

## Materials and methods

### Study design

This study was an observational cross-sectional study of 411 cull dairy cows transported to slaughter by truck in Denmark between January 2015 and 2016. All cows were examined clinically on farm before being loaded onto commercial trucks. After unloading at the slaughter plant, the clinical condition of the cows was re-examined. All clinical examinations before and after transport were done by the same veterinarian. Loading, transport, and unloading took place under conditions typical for commercial Danish cow transport. Transport distance, duration, and stops underway were recorded. One slaughter plant, four hauliers (with a total of five different trucks), and 20 dairy farmers participated in the study.

### Recruitment of participants

Dairy farmers were recruited via four hauliers transporting cull cows to the slaughter plant Danish Crown Beef (6670 Holsted, Denmark). The hauliers were recruited with help from two large Danish transport organizations, ITD (Dansk Transport & Logistik, 1019 Copenhagen, Denmark) and DTL (International Transport Danmark, 6330 Padborg, Denmark). The recruitment procedure is described in detail by Dahl-Pedersen et al. (9).

### Selection of cows

The participating farmers decided which cows to cull and followed their normal culling routines. The study did not include cows clearly unfit for transport. However, in order to include animals that were maybe fit for transport (in addition to cows fit for transport) in the study, an ethical permit was issued from the Animal Experiments Inspectorate (permit no. 2015-15-0201-00716), allowing the inclusion of animals of varying levels of fitness, as long as they did not classify as unfit with respect to the specific requirements of the EU regulation [EC 1/2005 (20)]. Following normal routines, the participating farmers contacted the slaughter plant directly in order to make arrangements for transport. The project veterinarian, in collaboration with either the farmer or the haulier, then decided if a transport could be included in the project with regards to the overall logistics and time schedule.

### Clinical examination on-farm

The clinical examination focused on locomotion, presence of wounds, milk leakage, and general condition (Table 1). Additionally, other aspects of the clinical condition of the cows were evaluated to identify cows unfit for transport and to gain information about possible risk factors for deterioration of the clinical condition during transport. These included e.g., rectal temperature, heart rate and respiratory frequency, body condition score (BCS), inspection/palpation of skin, hair coat, limbs, body, and udder, evaluation of peripheral and central circulatory system (including auscultation of heart), and evaluation of respiratory system (including auscultation of lungs) [details described in Dahl-Pedersen et al. (9)]. Pelvic asymmetry and digital dermatitis were scored as present or absent based on a visual inspection of the cows. Only active stages of digital dermatitis were recorded as “present” whereas healed lesions were not recorded as “present.” The cows were restrained in headlocks while examined, but let loose for locomotion scoring and observed walking a short distance of 5–10 m (22). The majority of cows were scored on slatted or solid concrete floors, and 8% were scored while walking in deep straw bedding. The clinical examination lasted ~5 min per cow. If the cows were to be loaded in the morning, they were examined clinically at the farm the same day. If the cows were to be loaded during the night, they were examined clinically the evening before. Until loading, the cows would either wait in a small pen inside the barn (17 farms) or wait in a small pen outside the barn (three farms). Depending on the routines of the farmers, the cows would wait in the pens from <30 min up to 8 h. No recordings were made during this period.

**Table 1:** Definitions for categorizing the clinical variables of the cull dairy cows before and after transport to the slaughter plant as normal or deviating from normal.

**Clinical condition**	**Definition of deviating from normal**
General condition	Not bright, alert, and responsive
Lameness	Locomotion score 3 or higher (22)
Wound	Lesion penetrating all layers of skin, size at least 1 × 1 cm
Milk leakage	Milk continuously dripping or flowing from one or more teats

### Transport to slaughter and associated recordings

**Figure 1:**
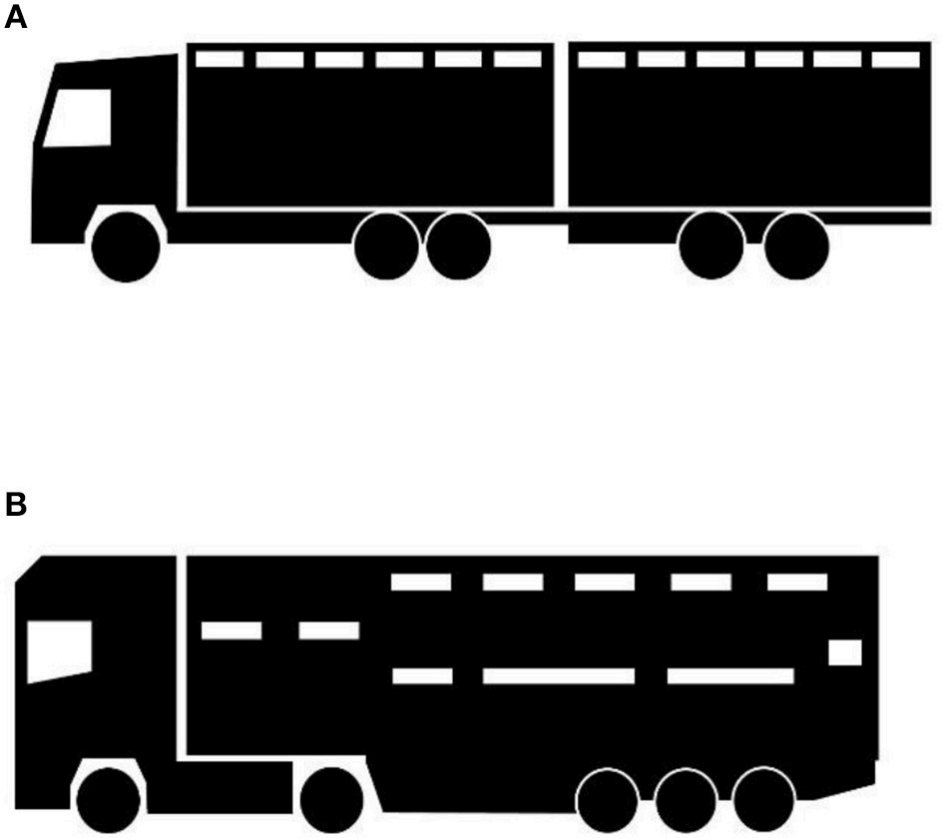
Schematic drawings of the two types of vehicles involved in the study: single deck truck with trailer **(A)**, and double deck truck **(B)**. During the study, cows were never transported on the upper deck. The white rectangles mark openings in the walls of the truck for ventilation.

All trucks used in the study were approved for transport of cows and all livestock drivers were authorized to transport cows according to Danish legislation. Four of the five trucks used in the study were single deck with trailer; one was double deck and no trailer (Figure 1). Single deck trucks with a trailer could carry a maximum of 25 cows, the double deck truck carried a maximum of 35 cows on the lower deck; cows were never on the upper deck in the present study. Current rules regarding space allowances during transport state that a cow with an approximate live weight of 325 kg must have 0.95–1.3 m^2^, a cow with an approximate live weight of 550 kg must have 1.3–1.6 m^2^, and cows with a live weight >700 kg must have >1.6 m^2^ [EC 1/2005 (20)]. These rules were complied with at all times. In all trucks, the floors were rubber-coated and sawdust was used as bedding.

The ramps were coated with rubber, fitted with foot battens and the slope could be adjusted to fit the surroundings when loading and unloading cows, but was never steeper than ~26° cf. the EU regulation [EC 1/2005 (20)]. The ramps were provided with side protections with an approximate height of 130–140 cm to avoid cows escaping or falling off. The trucks were passively ventilated through openings in the upper part of the side walls in order to ensure adequate ventilation above the cows when standing, cf. the EU regulation [EC 1/2005 (20)]. The ventilation openings were rectangular, with an approximate height of 20–25 cm and of varying length, but in total constituting < 10% of the wall area. All trucks had full air suspension. When loading, the truck drove up close to the barn and the cows were loaded directly from the small pen inside the barn or from the outside pen. The driver would walk behind the cows, hold a lightweight plastic board in front of him and gently drive the cows up the ramp and onto the truck. Sticks or electric pods were never used. At two farms, the cows were led by halter one at a time. The loading time did not exceed ~5 min.

For all journeys, the distance, duration, number of stops, and the duration of stops were recorded. A stop was defined as the truck not moving (irrespective of the reason) for at least 5 min. In Denmark, cull dairy cows are seldom shipped from one farm in great enough numbers to fill a whole truck, and hence, it is normal practice for hauliers to pass by other farms on the way to the slaughter plant and collect cows there. During journeys, drivers might shift the partitions between the cows inside the truck and move cows around as more cows are loaded onto the vehicle. Hence, it was not possible to register the precise stocking density or the occurrence of mixing of cows after loading. If a farmer had ordered transport with special provisions i.e., segregating a cow in order to protect her from the rest of the animals, this was maintained throughout the journey and the use of segregation was recorded.

### Clinical examination at the slaughter plant

Upon arrival at the slaughter plant, the waiting time until unloading was typically short, but delays inside the slaughter plant prolonged the waiting time on a few occasions (median 5 min, range 0–65 min). As the truck was opened and the partitions removed, the driver would enter the truck and gently move the cows, at their own pace, down the ramp from the truck. Unloading took <5 min. The cows were then separated into a holding pen, where they could move freely. The second clinical examination took place while the cows were in the holding pen and included a visual inspection of general condition, locomotion, wounds, and milk leakage. Definitions of clinical conditions are shown in Table 1.

### Additional data from the danish cattle database

As a supplement to data from the clinical examinations, data on age, parity, DIM, milk yield, and veterinary treatments during the past 6 months before culling were obtained from the Danish Cattle Database. The database is run by SEGES, a large Danish agricultural industry organization (SEGES, 8200 Aarhus N, Denmark), and holds detailed information entered by farmers, veterinarians, hoof trimmers, dairies, slaughter plants, and others involved in dairying in Denmark.

### Data analysis

Data were analyzed in SAS (version 9.4, SAS Institute Inc., Cary, NC). McNemar's test (PROC FREQ) was used to evaluate differences in the proportion of cows with a given clinical finding before and after transport. Deterioration of locomotion was defined as any increase in locomotion score, except for an increase from 1 to 2 (as both score 1 and 2 are considered not lame). For the other clinical findings evaluated before and after transport (wounds, milk leakage, and general condition), deterioration was defined as changes from absent to present. Cows with a clinical sign (lameness, a wound, milk leakage, or abnormal general condition) both before and after transport were thus recorded as having no deterioration.

Three groups of explanatory variables were included in an analysis of risk factors for deterioration of clinical findings. The first group focused on the clinical condition of the cows before transport. Factors related to the journey such as number of stops or duration of transport were treated as a second group. The third group of variables were related to the production of the cows and consisted of data such as parity, DIM, and milk yield retrieved from the Danish Cattle Database. No cows had a clinically abnormal general condition, neither before nor after transport. Risk factors for a deterioration of clinical findings were identified for each of the remaining outcomes: locomotion, wounds and milk leakage. The following step-wise procedure was used: In step 1, the association between the outcomes and explanatory variables from all three groups of explanatory variables were then screened one by one using a univariable logistic regression model (PROC GLIMMIX). Only variables with a *P*-value ≤ 0.25 were further analyzed in step 2, where the association between each of the outcomes and variables from step 1 with *P* ≤ 0.25 was evaluated using a binary logistic regression model (PROC GLIMMIX). The model was reduced using backwards elimination. All possible two-way interactions were included in the model. Confounding was evaluated comparing odds ratios with and without the possible confounder included in the model. If the difference was larger than 20%, important confounding was considered to be present. To evaluate the possible effect of cows at the same journey being more equal than cows at different journeys, journey was included as a random effect in the models. However, this only affected the results very little. The overall fit of the model was tested by evaluating the dispersion parameter which should be close to 1.

## Results

### Status of included cows

The study included a total of 411 cull dairy cows. Twelve cows, assigned for culling by the farmers, could not be included in the study as they were assessed as unfit for transport during the clinical examination at the farm. The cows were unfit due to severe lameness (score 5), fever, spastic paresis, and BCS < 2. These cows were left on farm and not included in this study.

The cows were culled after a mean of 2.9 lactations (range 1–10 lactations) and a mean of 270 DIM (range 15–871 days). The cows had a mean BCS of 3.25 (range 2.0–5.0) and were of the following breeds: Danish Holstein (68%), Red Danish Dairy (14%), Danish Jersey (8%), and crossbreeds (10%). The farmers stated the primary reason for culling: 28% of the cows were culled due to reproductive failure, 26% due to low milk yield, 15% due to udder health, and 13% due to lameness. The remaining 18% of the cows were culled due to a variety of other reasons.

### The transport to the slaughter plant

A total of 49 journeys were included. The journeys covered a mean distance of 129 km (range 20–339 km). Thirty-eight per cent (156/411) of the cows were transported < 101 km, 42% (173/411) were transported between 101 and 200 km, and 20% (82/411) were transported more than 200 km. The mean duration of journeys was 187 min (range 32–510 min). The study was designed to include journeys of up to 8 h (480 min), which is the legal maximum for transport of cull cows in Denmark, but due to a delay at unloading at the slaughter plant, six cows experienced a journey of 8.5 h (510 min). Forty percent of the cows (164/411) were transported for < 121 min, 35% (144/411) were transported between 121 and 240 min, and 25% (103/411) were transported for more than 240 min, including the six above-mentioned cows. The median number of stops underway was 2 (range 0–6 stops) and the median total duration of stops (including journeys with no stops) was 48 min (range 0–155 min). In five cases, a cow was transported with special provisions i.e., segregated from the other cows for protection.

### Fitness for transport

Nine cows arrived at the slaughter plant in a condition, where they, according to the current EU regulation [EC 1/2005 (20)], may have been judged as unfit for transport. They had all been lame (score 3 and 4) when leaving the farm and were severely lame (score 5) upon arrival.

### Clinical variables

**Figure 2:**
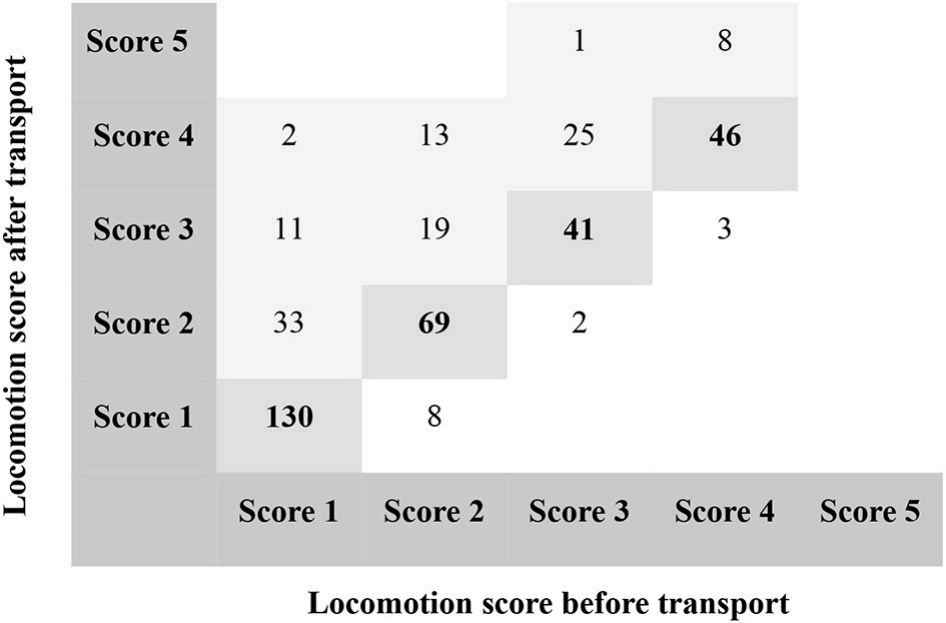
Number of cull dairy cows with a given locomotion score before (x-axis) and after transport (y-axis) to slaughter, *N* = 411. The diagonal line of numbers in bold represents cows that scored the same before and after transport. Numbers above the diagonal represent cows that scored higher after transport; numbers below the diagonal represent cows that scored lower after transport.

Three variables changed significantly during transport: locomotion score, milk leakage, and wounds. Significantly more cows were lame after transport than before (41% after vs. 31% before, *P* < 0.0001). Overall, 19% of the cows became lame or more lame during transport. Among cows that were not lame before loading (score 1–2) 15.8% became lame. Among cows that were lame before loading (score 3–4), 26.6% became more lame. Locomotion scores before and after transport is presented in Figure 2. Results from the logistic regression showed no association between factors related to the journey itself, i.e., distance, duration, number of stops underway, and duration of stops underway, and deterioration of locomotion scores during the transport. Deterioration of locomotion was significantly associated with production related factors and the clinical condition of the cow: early lactation (<100 DIM) and late lactation (>300 DIM), low BCS (< 2.75), digital dermatitis at the hind feet, and pelvic asymmetry (Table 2).

**Table 2:** Results from a logistic regression evaluating risk factors for deterioration of locomotion score during transport of 411 cull dairy cows to slaughter.

**Risk factor**	**Odds ratio**	**95% Confidence interval**	***P*-value**
Lactation stage			0.01
Early lactation (< 100 DIM)	1.9	0.9–4.1
Mid lactation (100–300 DIM)	1	
Late lactation (>300 DIM)	2.6	1.3–5.1
Body condition score			0.001
≤ 2.50	3.7	1.7–7.9
2.75–3.75	1	
≥4.00	0.7	0.3–1.4
Digital dermatitis at the hind feet			0.01
No	1	
Yes	3.0	1.6–5.7
Pelvic asymmetry			0.001
No	1	
Yes	4.9	1.4–16.3

Significantly more cows had milk leakage after transport than before (17% after vs. 1% before, *P* < 0.0001). Milk leakage was significantly associated with lactation stage and transport distance. Cows that were transported more than 100 km and cows in early lactation (<100 DIM) had significantly higher odds of milk leakage (Table 3).

**Table 3:** Results from a logistic regression evaluating risk factors for deterioration of milk leakage during transport of 411 cull dairy cows sent to slaughter.

**Risk factor**	**Odds ratio**	**95% Confidence interval**	***P*-value**
Lactation stage			0.04
Early lactation (< 100 DIM)	2.9	1.3–6.6
Mid lactation (100–300 DIM)	1.3	0.7–2.6
Late lactation (>300 DIM)	1	
Distance of journey			0.006
>200 km	10.2	2.2–46.9
101–200 km	8.6	2.1–34.4
< 101 km	1	

Significantly more cows had wounds after transport than before (34% after vs. 22% before, *P* < 0.0001). A total of 103 new, bleeding wounds were recorded together with nine older wounds where the scab covering the wound had been torn off during transport. The new wounds were primarily found on the fetlock, hock, and hips. None of the risk factors included in the analysis were significantly associated with the increase in proportion of cows with wounds.

## Discussion

This observational study is among the first to compare selected clinical signs of cull dairy cows before and after transport to slaughter. The objectives of the study were to investigate whether the clinical condition of the cows deteriorated during commercial transport of up to 8 h in duration as well as to evaluate possible risk factors for such a deterioration. In our dataset, including only cull dairy cows which were not unfit for transport while on-farm according to the EU regulation [EC 1/2005 (20)], one-fifth of the cows became either lame or more lame during transport and a significant increase in the proportion of cows with milk leakage and wounds were found after transport. A number of risk factors for lameness and milk leakage was identified, whereas no risk factors for wounds could be identified. The results provide a basis for a deeper understanding of the consequences of transport for cull dairy cows, showing increased occurrence of injuries after transport in cull cows legally considered fit for transport. These results call for further research into the animal welfare implications and optimization of cattle transport, possibly also including intervention studies where effects of single factors can be investigated.

The identified risk factors were primarily related to the individual cow (her clinical condition and production related factors), but surprisingly only to a smaller extent to characteristics of the journeys such as duration or number of stops. In the current EU regulation [EC 1/2005 (20)], especially duration of transport is an important parameter, used to define what requirements should be met e.g., in terms of stocking density, feeding, and resting during transport. Legally, journeys are split into short (<8 h) and long journeys and regulated accordingly. The present study focused on journeys of up to 8 h. Studies of journeys of a longer duration than 8 h have shown that duration can have adverse effects on the clinical condition of cattle. Knowles et al. (23) investigated journeys of fattening cattle of different durations between 14 and 31 h and found that levels of cortisol increased with transport time and that some of the animals showed signs of fatigue and lay down after 20 h. Somewhat similar, Gonzalez et al. (12) found that on long haul journeys, the longer the cows were on the truck, the greater the risk of becoming lame, non-ambulatory, or dying. However, the present results suggest that in the case of shorter journeys of cull dairy cows, the duration is not a major risk factor for changes in the clinical condition of the animals. Further research is needed in order to examine effects of journey duration on cull dairy cows when transported more than 8 h, as is practice in many countries other than Denmark (1).

As the present study did not include any comparison between cull dairy cows and other types of cattle, it cannot be confirmed directly that cull dairy cows are more vulnerable to the stress of transport than other types of cattle as previously has been suggested (10, 13, 16). However, the present results do show that transport (even shorter than 8 h) is a straining experience for cull dairy cows. A considerable proportion of the cows were injured in terms of lameness or wounds, which is a violation of the EU regulation [EC 1/2005 (20)], clearly stating that cows must not get injured during transport. Yet, these cows would not be considered unfit according to the same EU regulation [EC 1/2005 (20)], thus the legal implications of the injuries that occurred during transport remain unclear.

The large proportion of cows that became lame or more lame during transport in the present study is in contrast to results from a study by Thomsen and Sørensen (15), showing that short distance transport of dairy cows (mean 84 km, 115 min) did not result in changes in locomotion score. However, the cows in the study by Thomsen and Sørensen (15) were all non-lame lactating dairy cows not due to be culled. Hence, despite the lack of a direct comparison, and differences in study design (experimental vs. observational) and distance driven (mean 84 vs. 129 km), the results from the present study may indicate that cull dairy cows are more vulnerable to the stress of transport than dairy cows not due to be culled. However, studies involving direct comparisons are needed to clarify this. In the present study, a few cows (13 out of 411) had a lower locomotion score after transport than before transport. This is likely due to an effect of the observer rather than a truly better locomotion after transport (22).

A small proportion of the cows (8%) were locomotion scored before transport in holding pens with deep straw bedding. Different floor types may influence the locomotion score of cows and softer surfaces can have a positive impact on the gait (24, 25). This may have meant that cows scored on deep straw bedding would have had a systematically lower locomotion score before transport compared to cows scored on concrete. However, cows scored on straw before transport did not become more lame when scored on concrete at the slaughter plant compared to cows scored on concrete both before and after transport (data not shown). The reason for this is perhaps that the cows had not been housed permanently on deep straw bedding prior to transport and therefore they were used to walking on hard surfaces.

We found that the risk factors for becoming lame or more lame included low BCS and pelvic asymmetry. Both these clinical conditions can be recognized without having to perform a thorough clinical examination. Earlier studies including our own data from pre-transport (9) have linked BCS to lameness: (26) did an 8-years study of one dairy herd and found that low BCS predisposed cows to lameness and that the risk of lameness decreased with increasing BCS. Similarly, (27) found that cows with low BCS were more likely to be treated for lameness during the following 4 months. Thus, future recommendations for assessment of fitness for transport of cull dairy cows could include that special attention should be given to cows with low BCS.

Pelvic asymmetry can be caused by different severe pathological conditions, such as coxofemoral luxation or fractures of the pelvis which may result in varying degrees of lameness (28). Pelvic asymmetry and lameness thus may share common etiologies and the associations between the two conditions are not entirely clear: Does pelvic asymmetry cause lameness, does lameness cause pelvic asymmetry, or are the two conditions simply caused by the same underlying pathology? How cows with pelvic asymmetry are affected by transport has to our knowledge not been investigated before, but a statement from the Danish Veterinary Health Council advises against transporting cows with pelvic asymmetry and/or unspecific lameness of the hind legs as it may cause the cows undue suffering (29). However, further research is needed in order to understand the welfare consequences of pelvic asymmetry in general, and in relation to transport specifically. It needs to be clarified whether cows with pelvic asymmetry can be transported with special provisions without risk of further injury or increased strain, or if cows with pelvic asymmetry should simply not be transported.

The present study showed a significant increase in the proportion of cows with milk leakage after transport compared to before. In the current EU regulation [EC 1/2005 (20)], milk leakage is not associated with reduced fitness for transport. Yet, overfilling of the udder has been related to discomfort or pain. A study by Bertulat et al. (30) showed higher udder pressure after sudden dry-off as well as elevated levels of fecal glucocorticoid metabolites, interpreted as an indirect indicator of stress related to pain. Similarly, Kohler et al. (31) investigated the potential effects of a prolonged milking interval (24 h) and found increased udder pressure, decreased eating time as well as changes in locomotion such as an increased abduction of the hind legs, possibly to avoid pressure on the udder. In the present study, the risk factors for milk leakage were early lactation (< 100 DIM) and distance transported (>100 km). The cows that were transported for the relatively long distances typically came from farms, where the loading took place in the middle of the night. In many cases, the cows had been milked in the evening as part of the normal farm routine, and then waited for several hours to be loaded and transported. Contrarily, cows from farms closer to the slaughter plant would normally be milked in the morning right before being loaded. Thus, the effect of distance might be an effect of time since last milking. If so, it is not only the time cows spend on the truck that matters, but the entire interval from last milking to slaughter. This underlines the importance of milking the cows immediately before loading in order to reduce milk leakage and the potential pain or discomfort related to overfilling of udders. This is already addressed in the EU regulation [EC 1/2005 (20)], where is it stated that lactating animals must be milked immediately before loading and then at intervals of not more than 12 h. Additionally, the daily milk yield may also play a role for the risk of milk leakage after transport. High yielding cows may have a higher risk of milk leakage after transport. This risk may be reduced by decreasing the number of daily milking gradually a few days before transport to slaughter. Future research on milk leakage and the related discomfort or pain should include precise information of time from last milking to slaughter to elucidate this matter further.

None of the wounds recorded after transport in the present study were considered “severe,” as is listed as a specific reason for a cow to be judged as unfit for transport in the current EU regulation [EC 1/2005 (20)]. However, as mentioned above, the regulation does state that cows must not be transported in a way that causes injury and in that sense the present wounds were violations of the regulation. The presence of the injuries shows that transport conditions were sub-optimal. None of the risk factors included in the study were, however, associated with the increasing proportion of wounds, and further studies are required to determine how, why and when the wounds appeared and how to avoid them. Strappini et al. (32) investigated bruising events in cull cows and found rough handling during loading and unloading, and inadequate stunning facilities to be the most important risk factors. These risk factors cannot explain the increase in the proportion of wounds found in the present study, however, as rough handling was never observed and the clinical examinations were done before the cows entered the stunning box. Very little is known about what goes on inside the trucks during the different stages of transport and more knowledge is needed. Cockram and Spence (33) used video recordings to monitor trucks transporting cattle in order to evaluate effects of driving events on the stability of the animals. They found that cattle primarily lost the balance during cornering and that losses of stability were five times more frequent on minor and main roads compared to motorways. Further studies, including collection of behavioral data during journeys, including both driving and stationary periods as well as loading and unloading are needed to provide information about where in the pre-slaughter logistic chain injuries might be inflicted and, subsequently, avoided.

All clinical observations in the present study were made by the same trained veterinarian. Even though this design removes the risk of inter-observer disagreement, the observer might remember the pre-transport scores of a particular cow and may be biased by this during the after transport scoring of the cow. However, we find that this bias is small and insignificant in the present setting.

## Conclusion

The objectives of this observational study were to determine whether the clinical condition of cull dairy cows deteriorate during commercial transport of up to 8 h duration as well as to evaluate possible risk factors for such a deterioration. The study revealed a significant deterioration of lameness, milk leakage and number of wounds. A number of risk factors for lameness and milk leakage were identified, mainly related to the individual cow (her clinical condition and production related factors), and to a minor extent to characteristics of the journey such as the distance. No risk factors for wounds could be identified. Overall, the results indicate that future recommendations for assessment of fitness for transport of cull dairy cows could specify that special attention should be given to cows with low BCS, cows showing signs of lameness before loading, cows in early or late lactation, cows with digital dermatitis and cows with pelvic asymmetry. Lactating animals should always be milked immediately before loading. The observed increased occurrence of injuries after transport in cows legally considered fit for transport, calls for further research and development into the concept of fitness for transport as well as a consideration of the implications for animal welfare and strategies that would optimize transport of cattle.

## Author contributions

PT and MH conceived the idea of the study. The experimental protocol was developed by all authors. KD-P collected all data and did the data analyses together with PT. KD-P did the literature search and drafted the manuscript, which was edited and finalized by all authors.

### Conflict of interest statement

The authors declare that the research was conducted in the absence of any commercial or financial relationships that could be construed as a potential conflict of interest.
